# A Meta-Analysis of the Protective Efficacy of *Brucella abortus* S2308 Gene-Deletion Mutant Vaccines Compared to Conventional Vaccines in Mice

**DOI:** 10.3390/microorganisms14071419

**Published:** 2026-06-29

**Authors:** Jing Yuan, Maoyuan Gu, Rongrong Ni, Xufeng Liu, Qimin Dong, Mengdi Dong, Shuqi Dong, Zeyao Wang, Tianyue Zhang, Yuting Zhang, Zhujie Fu, Junyuan Li, Yanbing Zhang, Huan Zhang, Zhongchen Ma, Junbo Zhang, Jihai Yi, Yueli Wang

**Affiliations:** 1 College of Animal Science and Technology, Shihezi University, Shihezi 832003, China; 18700796620@163.com (J.Y.); g_summery@163.com (M.G.); 15105582511@163.com (R.N.); qimindong2026@163.com (Q.D.); 17716950349@163.com (M.D.); 13040546919@163.com (S.D.); 18105616208@163.com (Z.W.); 15609932430@163.com (T.Z.); 18838055832@163.com (Y.Z.); f15840888775@163.com (Z.F.); 19176800010@163.com (J.L.); zhangyanbing@shzu.edu.cn (Y.Z.); zhanghuan@shzu.edu.cn (H.Z.); zhongchen_ma@163.com (Z.M.); 2 School of Clinical Medicine, Shihezi University, Shihezi 832003, China; l1170515xf@163.com; 3 Guizhou Provincial Key Laboratory for Biodiversity Conservation and Utilization in the Fanjing Mountain Region, Tongren University, Tongren 554300, China; zhangjunbo666@126.com

**Keywords:** *Brucella*, gene-deletion mutants, conventional vaccines, protective efficacy, meta-analysis

## Abstract

Brucellosis remains a major zoonotic disease that affects both public health and animal production worldwide. Conventional live vaccines, including S19, RB51, and A19, are commonly used for disease control; however, concerns regarding their safety and performance under field conditions limit their application. Targeted gene deletion in *Brucella abortus* S2308 has generated multiple candidate vaccine strains, but their protection relative to conventional vaccines has not yet been clearly established. We synthesized evidence from 15 mouse studies to compare the protective performance of S2308-derived gene-deletion mutants with that of conventional vaccines. The pooled estimate did not show a statistically significant difference in splenic bacterial burden after challenge between mutant and conventional vaccine groups (mean difference [MD] = 0.38, 95% CI: −0.07 to 0.84; *p* = 0.10), although substantial heterogeneity was observed (I^2^ = 95.38%). Heterogeneity was partially explained by mouse strain, mouse age, the functional categories of deleted genes, number of deletions, and challenge dose. Mutants targeting signal-regulatory genes tended to be associated with lower splenic bacterial loads than those targeting genes involved in lipopolysaccharide (LPS) biosynthesis. Overall, based on post-challenge splenic bacterial burden, current mouse evidence suggests that S2308-derived gene-deletion mutants may reduce bacterial burden to an extent broadly comparable to that achieved by conventional *Brucella* vaccines in mice. The observed association between signal regulation-related gene deletions and lower splenic bacterial burden should be regarded as exploratory and requires further validation using broader protective endpoints and studies in natural target hosts.

## 1. Introduction

Brucellosis, transmitted between animals and humans, continues to impose considerable burdens on livestock production and public health [1]. In affected cattle, the disease may cause reproductive disorders, including abortion, infertility, impaired reproductive performance, and reduced milk yield [2,3]. Although comprehensive control measures have substantially reduced disease occurrence in many developed settings, bovine brucellosis continues to circulate in several regions of Asia, Africa, and the Americas [4]. Experimental brucellosis studies commonly use *Brucella abortus* 2308 as a reference challenge strain; this isolate was originally obtained from an aborted bovine fetus and exhibits a stable, reproducible virulence phenotype [5].

Prevention programs for bovine brucellosis have traditionally depended on live attenuated vaccines, particularly S19 and RB51 [3]. Despite their utility, both vaccines have recognized limitations, including residual virulence, vaccine-associated abortion during gestation, potential zoonotic infection, and occasional persistence of vaccine strains in immunocompromised hosts [6,7]. These constraints have driven continued efforts to develop safer and more effective vaccines. Recent vaccine-development strategies have combined omics-based insights, such as genomics and proteomics, with genetic manipulation approaches, including recombinant DNA technologies [8,9]. Within this context, rationally attenuated vaccines generated by targeted gene-deletion strategies remain a major focus because attenuation can be more precisely controlled while retaining strong immunogenicity [10]. Several engineered *Brucella* gene-deletion vaccine candidates have been reported, including A19-Δ*VirB12* and several additional engineered mutants, supporting the translational potential of genetically engineered live vaccines [11,12].

S2308-derived gene-deletion mutants have been evaluated in multiple mouse challenge studies. However, direct comparison across studies is difficult because of substantial heterogeneity in animal models, immunization regimens, challenge conditions, and deletion strategies. In addition, the protective efficacy of these mutants relative to conventional vaccines has not been quantitatively synthesized, and it remains unclear whether deletion of different functional gene categories is associated with differences in protection. Therefore, we conducted a meta-analysis of mouse studies on S2308-derived gene-deletion mutants to compare their protective efficacy with that of conventional vaccines and to explore whether deleted gene function may influence vaccine performance.

## 2. Materials and Methods

The reporting of this meta-analysis followed PRISMA recommendations. The protocol was registered in PROSPERO before study selection began (CRD420251108038). The completed checklist is available through Figshare at https://doi.org/10.6084/m9.figshare.30926654.

### 2.1. Search Strategy

Records were retrieved from PubMed, Web of Science, ScienceDirect, CNKI, Wanfang Data, and VIP without applying a lower date limit, and the search was updated through 31 December 2025. The query terms were organized around *Brucella*, *B. abortus* S2308, gene-deletion mutants, vaccination, and protective efficacy. The complete database-specific search strategies are reported in Table S1.

### 2.2. Inclusion and Exclusion Criteria

Studies were eligible when they met all of the following requirements: (1) study subjects: mouse models, with no restriction on strain, sex, or age; (2) intervention: inoculation with a clearly identified S2308-derived gene-deletion mutant, with the deleted gene explicitly specified; (3) comparator: conventional vaccine strains (S19, RB51, or A19); and (4) outcome measures: post-challenge splenic bacterial load quantified as log_10_ colony-forming units (CFUs), with extractable numerical data available.

Records were removed for any of the following reasons: (1) inappropriate publication types, including reviews, commentaries, conference abstracts, and case reports; (2) studies that lacked in vivo animal challenge experiments; (3) insufficient data reporting, in which splenic bacterial load was presented only graphically without retrievable numerical values; (4) inaccessible full texts or publications in languages other than English or Chinese; and (5) duplicate reports, in which case the most complete dataset was retained.

### 2.3. Data Extraction

Before data collection, a structured worksheet was prepared for recording information from the eligible reports. Each included study was reviewed separately by two reviewers. Recorded information included publication details, mouse strain and age, gene-deletion name and functional category, immunization and challenge parameters, and post-challenge splenic bacterial load (log_10_ CFU). The extracted data were cross-checked between reviewers. Any inconsistency was discussed until agreement was reached; when necessary, a third reviewer made the final determination.

### 2.4. Risk-of-Bias Assessment

Two reviewers separately appraised the risk of bias in the included animal studies using the SYRCLE tool. This assessment considered bias arising from study selection, performance, outcome detection, attrition, selective reporting, and other possible sources. For each domain, judgments were assigned as low risk, high risk, or unclear risk in accordance with the SYRCLE guidance. Differences in assessment were resolved by reviewer discussion, with third-reviewer adjudication used when consensus could not be achieved.

### 2.5. Analysis Strategy

All statistical analyses were undertaken in Stata version 17.0. The primary effect measure was the mean difference (MD) in post-challenge splenic bacterial burden, expressed as log10 CFU, between the *B. abortus S2308*-derived gene-deletion mutant group and the conventional vaccine comparator group. Effect estimates were reported with 95% confidence intervals (CIs). The MD was calculated such that a value greater than zero represented a higher splenic bacterial load in mice receiving the S2308-derived mutant, whereas a value below zero indicated a lower bacterial load relative to conventional vaccine controls. Because the included comparisons showed substantial variability, pooled estimates were obtained with a random-effects model. The between-study variance was quantified as τ^2^ using restricted maximum likelihood (REML), and confidence intervals around pooled estimates were obtained with the Knapp–Hartung method. Heterogeneity was assessed by Cochran’s Q statistic, I^2^, and τ^2^; I^2^ values above 50% were interpreted as substantial heterogeneity. Potential contributors to heterogeneity were examined through prespecified subgroup analyses and univariable meta-regression. To assess the stability of the pooled effect, sensitivity analyses were performed by removing one study at a time. Small-study effects were evaluated through funnel-plot inspection and Egger’s regression test. When funnel-plot asymmetry suggested the possible influence of missing studies, the trim-and-fill procedure was applied. All statistical tests were two-sided, and *p* values below 0.05 were considered statistically significant.

## 3. Results

### 3.1. Screening Outcome

**Figure 1:**
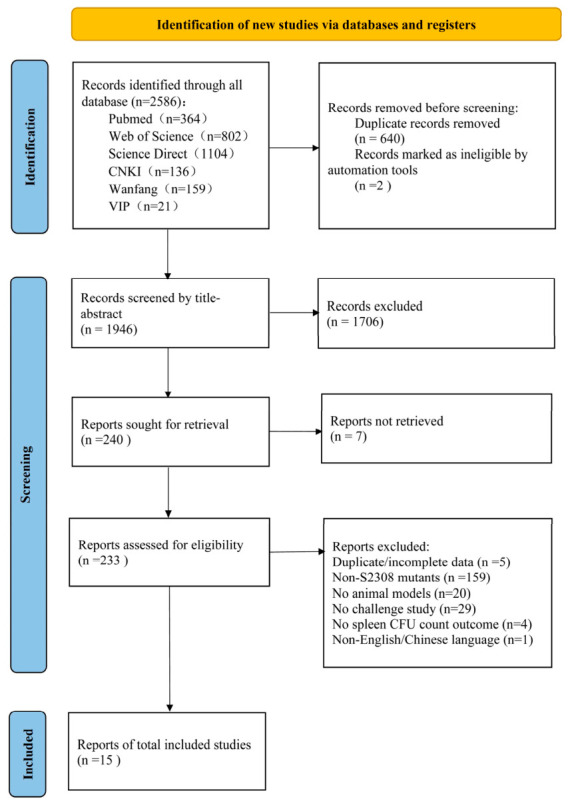
Record selection pathway for the included studies.

The initial database retrieval produced 2586 records. These records were progressively filtered by duplicate checking, title and abstract review, and full-text evaluation, leaving 15 studies for the meta-analysis. Figure 1 summarizes the number of records retained or excluded at each stage of the selection process.

### 3.2. Characteristics of Included Studies

In this study, each independent article was treated as a “study”, and each independent comparison between an S2308-derived gene-deletion mutant and a conventional vaccine control group within a single study was regarded as one “comparison group”. A total of 15 studies were included, comprising 28 comparison groups (Table 1). All studies used mouse models aged 4 to 9 weeks. The primary outcome measure in all studies was splenic bacterial load two weeks post-challenge, expressed as log_10_ CFU, and the method used to quantify splenic colony-forming units was consistent across studies.

### 3.3. Risk of Bias in Included Studies

**Figure 2:**
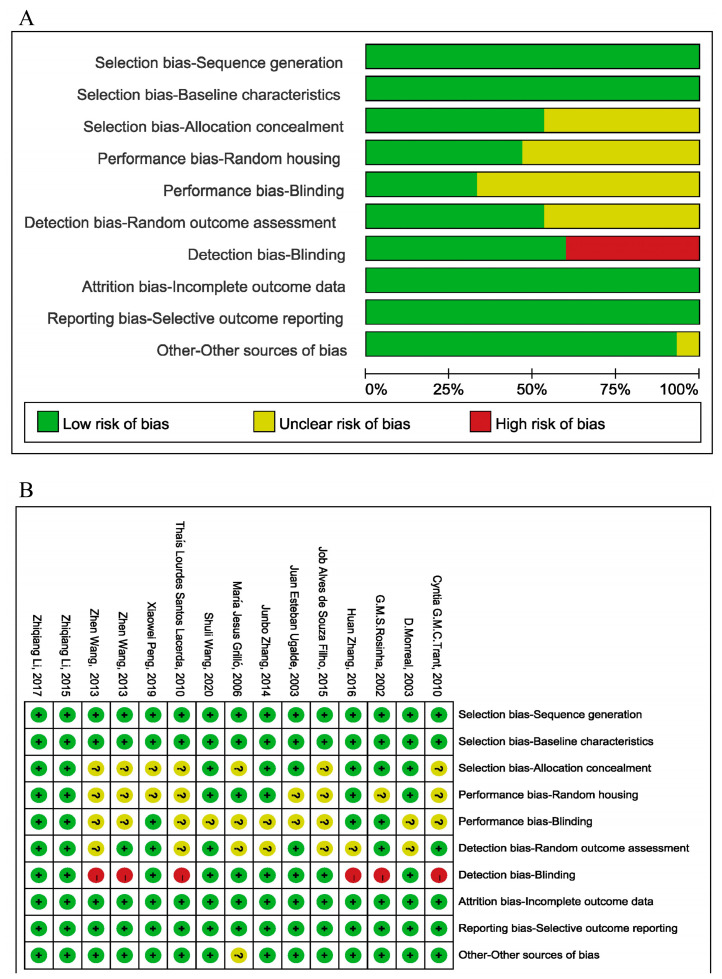
Results of the SYRCLE assessment. (**A**) Summary by assessment domain. (**B**) Ratings for individual included studies. In panel (**B**), “+”, “?”, and “−” indicate low, unclear, and high risk of bias, respectively [13,14,15,16,17,18,19,20,21,22,23,24,25,26,27].

The SYRCLE assessment results are summarized in Figure 2. Low-risk judgments predominated across the evaluated domains; however, some domains were classified as unclear or high risk. These uncertainties were mainly attributable to insufficient reporting in several primary studies, particularly with respect to randomization procedures, allocation concealment, blinding, and housing conditions. Therefore, although the included studies showed an overall acceptable risk-of-bias profile, incomplete methodological reporting limited the certainty of the assessment and precluded complete exclusion of potential bias.

### 3.4. Overall Pooled Effect

**Figure 3:**
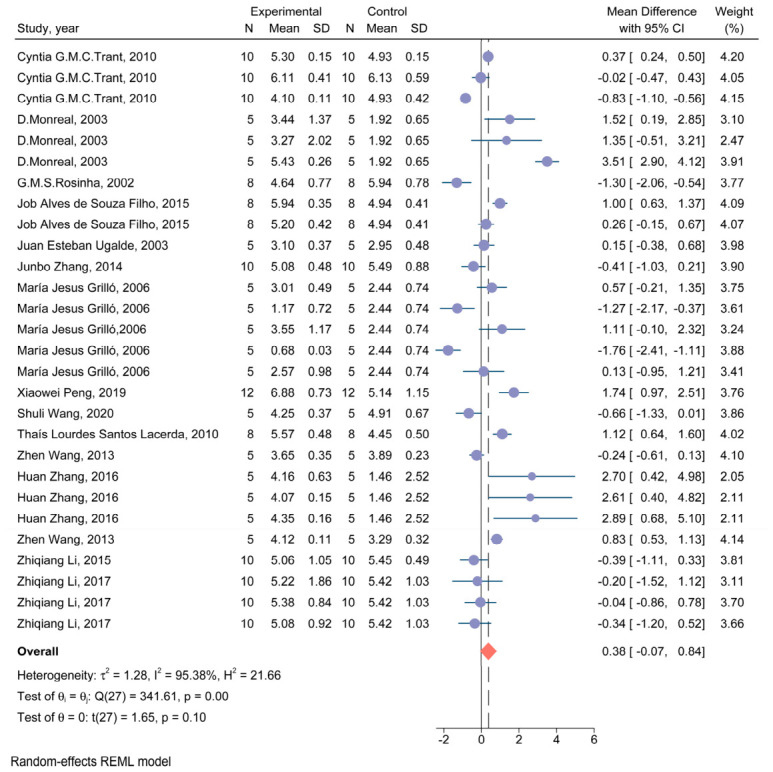
Forest plot comparing protective efficacy between S2308-derived gene-deletion mutants and conventional vaccines based on post-challenge splenic bacterial load, expressed as log10 colony-forming units (CFUs) [13,14,15,16,17,18,19,20,21,22,23,24,25,26,27].

A total of 15 studies contributing 28 independent comparisons were synthesized using a random-effects model. The analysis revealed no statistically significant difference in post-challenge splenic bacterial load between S2308-derived gene-deletion mutants and conventional vaccines (MD = 0.38, 95% CI: −0.07 to 0.84; *p* = 0.10). Within the limits of the available mouse data, this finding suggests that S2308-derived gene-deletion mutants may reduce splenic bacterial burden to an extent broadly similar to that achieved by conventional vaccine strains, as visually summarized in the forest plot (Figure 3).

### 3.5. Exploration of Heterogeneity

**Figure 4:**
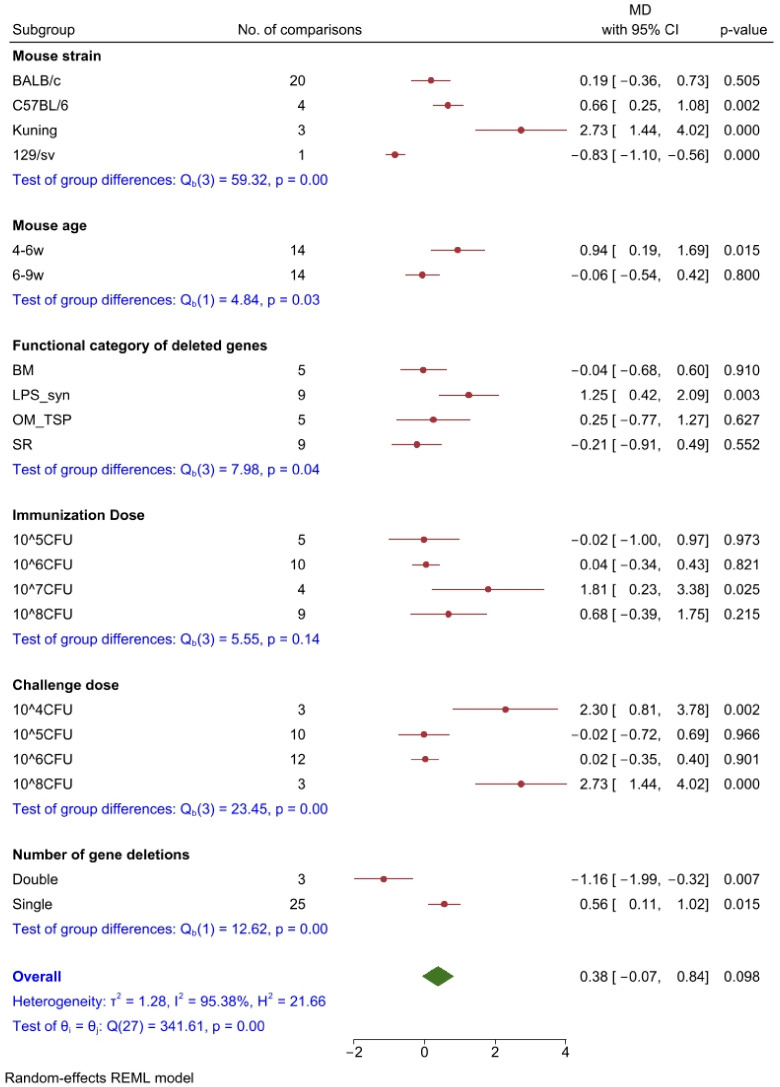
Forest plot of Subgroup analyses.

Substantial heterogeneity was observed in the overall analysis (I^2^ = 95.38%; Figure 3). Subgroup-specific pooled estimates were generated for the prespecified study characteristics to determine whether differences across categories accounted for part of the observed variation. Figure 4 presents the pooled estimates across the prespecified subgroups, whereas forest plots for the individual subgroup factors are provided separately in Figures S1–S6. Significant between-subgroup differences were observed for mouse strain, mouse age, functional category of deleted genes, number of gene deletions, and challenge dose, suggesting that these variables may contribute to the observed heterogeneity. However, considerable heterogeneity persisted within the subgroups (I^2^ > 70%), indicating that these variables alone did not fully explain the observed variability. Therefore, meta-regression analysis was further performed to identify potential contributors to between-study variation.

Given that substantial heterogeneity remained within several subgroups, univariable meta-regression was further performed to assess whether the subgroup variables identified above could explain between-study variation in effect estimates. The meta-regression results were generally consistent with the subgroup analyses, showing that mouse strain, mouse age, functional category of deleted genes, challenge dose, and number of deleted genes were significantly associated with variation in protective efficacy (Table 2). Among these variables, challenge dose explained the largest proportion of heterogeneity (R^2^ = 47.57%), followed by number of deleted genes (R^2^ = 18.77%), functional category of deleted genes (R^2^ = 17.75%), mouse strain (R^2^ = 14.25%), and mouse age (R^2^ = 10.55%). Overall, meta-regression provided additional support for the subgroup findings and suggested that differences in challenge conditions, gene-deletion characteristics, and mouse model-related factors contributed to variability in protective efficacy across studies.

### 3.6. Publication Bias and Sensitivity Analysis

**Figure 5:**
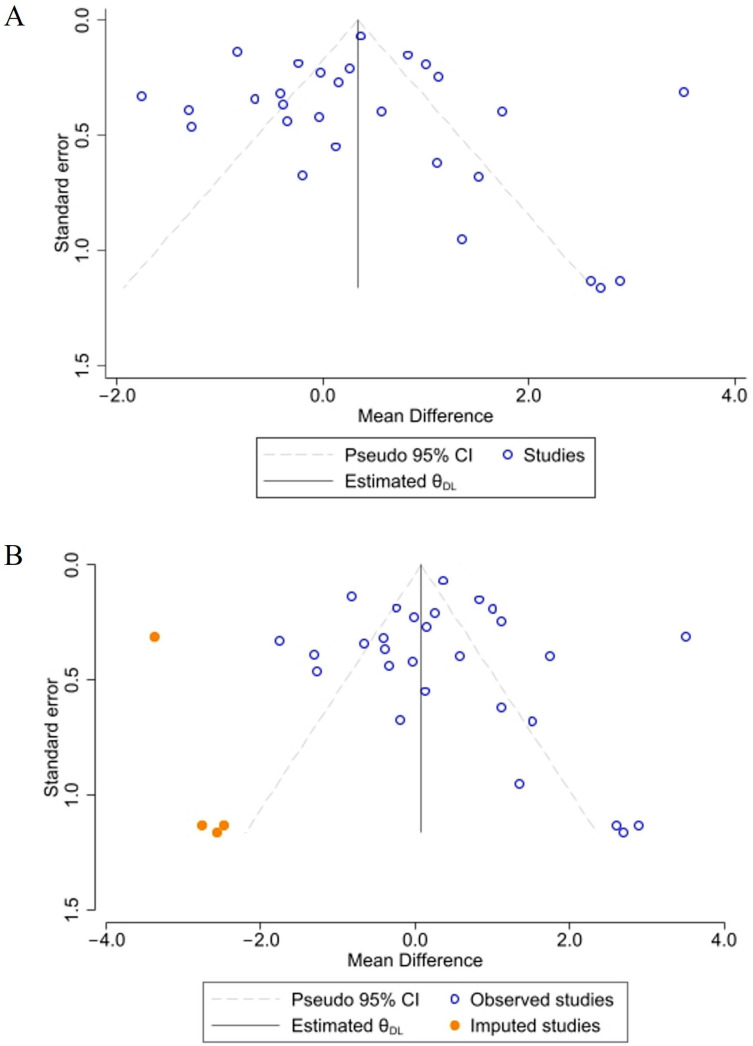
Funnel-plot results before and after trim-and-fill adjustment. (**A**) Original funnel plot for the included comparison groups; (**B**) Funnel plot after imputation of potentially missing studies.

**Figure 6:**
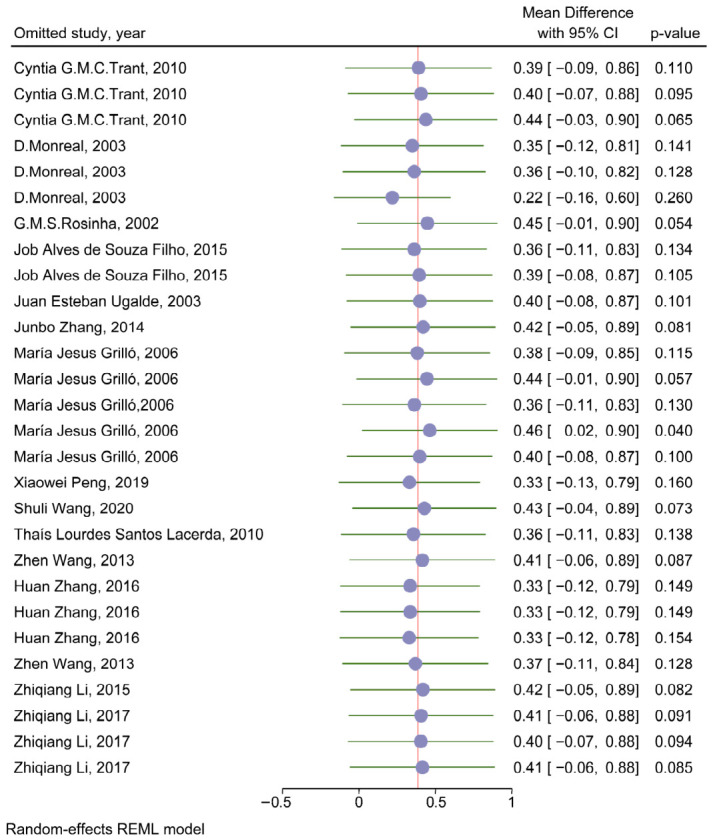
Sensitivity analysis of the pooled effect size [13,14,15,16,17,18,19,20,21,22,23,24,25,26,27].

Study distribution in the funnel plot was not symmetrical (Figure 5A), and Egger’s regression suggested small-study effects (*p* = 0.03). The trim-and-fill analysis imputed four potentially missing studies on the negative side, but the adjusted pooled estimate differed only minimally from the original estimate (Figure 5B). Sensitivity analysis based on one-study-at-a-time exclusion showed that the summary effect remained stable, suggesting that no single study drove the main result (Figure 6).

## 4. Discussion

As a zoonotic livestock disease, Bovine brucellosis generates considerable economic burdens through adverse effects on livestock health and productivity, together with additional public health-related costs associated with human infection [28,29]. In this context, rationally attenuated live vaccines based on targeted gene deletion in *Brucella abortus* have emerged as a promising strategy. Previous studies have demonstrated that deletion of different genes can attenuate bacterial virulence while maintaining or enhancing immunogenicity, thereby enabling the induction of protective host immune responses [30,31,32]. Nevertheless, the biological consequences of deleting individual genes are not uniform, and both the functional categories of the deleted genes and the associated immunization protocols are likely to influence vaccine performance. The present meta-analysis provides a quantitative synthesis of the available evidence comparing the protective efficacy of S2308-derived gene-deletion mutant vaccines with that of conventional vaccine strains in mouse models. Overall, the pooled results suggest that S2308-derived gene-deletion mutants may provide protective efficacy broadly comparable to that of conventional vaccines in mice. Accordingly, the pooled finding warrants cautious interpretation because substantial heterogeneity was present.

The substantial heterogeneity observed in this meta-analysis is biologically and methodologically plausible. The protective efficacy of live attenuated *Brucella* vaccines may be influenced by multiple interacting factors, including the functional role of the deleted gene, the number of deleted genes, the conventional vaccine strain used as the comparator, the challenge dose, and host-related characteristics such as mouse strain and age. These factors may affect the balance between attenuation and immunogenicity, the intensity of post-challenge infection, and the magnitude of host immune responses. Specifically, gene deletions affecting different biological pathways may produce distinct effects on bacterial survival, antigen exposure, and immune recognition; challenge dose may alter infection pressure and the sensitivity of vaccine efficacy assessment; and mouse strain or age may influence susceptibility to infection and the development of protective immunity. For instance, *Brucella* LPS contributes to immune evasion by limiting early innate immune recognition, whereas disruption of LPS structure can alter attenuation and host recognition [33]. Thus, the high heterogeneity observed across studies likely arose from both statistical variation and genuine differences in biological characteristics and experimental design.

Subgroup analyses and meta-regression identified several candidate contributors to the observed heterogeneity; however, these associations should be interpreted as hypothesis-generating rather than as confirmatory evidence. Residual heterogeneity remained after these analyses, indicating that the examined covariates did not fully explain the variability across studies. In addition, some subgroup comparisons included only a limited number of studies, which reduces statistical power and increases uncertainty in subgroup-specific estimates. Accordingly, the observed associations involving gene-deletion characteristics, challenge dose, and host-related factors should be interpreted as hypothesis-generating findings that require further validation in well-designed experimental studies using standardized protocols.

Exploratory subgroup and meta-regression analyses suggested that deletion mutants targeting signal regulation-related genes tended to show more favorable protective effects than those targeting LPS synthesis-related pathways, based on post-challenge splenic bacterial burden in mouse models. However, this observation should be interpreted cautiously because several functional categories were represented by limited numbers of studies or comparisons, and substantial residual heterogeneity remained. Therefore, the observed pattern remains preliminary and does not establish that deletions affecting signal-regulatory pathways yield greater protection. From a biological standpoint, variation in the functions of the deleted genes may underlie part of the marked heterogeneity in protective efficacy among the mutant strains. Alterations in LPS biosynthesis can shift *Brucella* from the smooth (S) to a rough (R) phenotype; although R-type strains generally display reduced virulence and a diminished capacity to evade host immune defenses, structural changes in LPS may also impair antigen presentation and thereby limit the induction of robust protective immunity [34,35,36]. Consequently, attenuation achieved primarily through disruption of LPS synthesis does not necessarily translate into strong or consistent vaccine-induced protection. In contrast, deletion of genes involved in signal regulation—such as the *bvrR*/*bvrS* two-component system—can directly interfere with multiple virulence-associated processes that are critical for intracellular survival. Deficiency of this system impairs host-cell invasion, inhibition of phagosome–lysosome fusion, and intracellular replication, while simultaneously altering the expression of outer membrane proteins (e.g., OMP22 and OMP25) and non-protein membrane components. These changes may disrupt outer membrane stability, increase antigen exposure, and enhance immune recognition [35,37]. Taken together, these biological considerations may help explain why signal regulation-related gene deletions showed a more favorable trend in the present analysis, but further standardized experimental studies are required to confirm whether these genes represent more advantageous targets for rational attenuation.

Beyond gene function, several experimental design-related factors also influenced efficacy estimates. Meta-regression suggested that double-gene-deletion strains generally provided stronger protection than single-gene deletion mutants; however, previous experimental studies have reported that multigene deletion does not necessarily result in superior protection in all contexts, indicating that the protective outcome depends on the specific combination and functional roles of the deleted genes [38,39]. This observation suggests that the benefit of multigene deletion is more closely related to the biological relevance and complementarity of the selected targets than to the number of deletions alone. With respect to immunization protocols, immunization dose was not significantly associated with protective efficacy in our analysis, although previous studies have suggested that vaccine dose may influence vaccine-induced protection [40]. This finding may be explained by the fact that most studies employed immunization doses within an effective “immune response plateau” that were sufficient to induce baseline protective immunity without inducing immune tolerance. In contrast, in our regression analyses, challenge dose emerged as an important moderator of efficacy. This suggests that deviations from the optimal dose range (2 × 10^5^ CFU) may influence infection intensity and compromise the accuracy of protective efficacy assessment [41].

Host-related factors also represent an important source of variability in protective efficacy. Across the included experiments, BALB/c mice were the most frequently represented model, whereas C57BL/6, Kunming, and 129/Sv mice were used in only a limited number of studies. Because only a small number of studies were conducted in certain mouse strains, conclusions based on these underrepresented models—particularly Kunming and 129/Sv mice—should be interpreted with caution. Well-documented immunological differences among mouse strains—including Th2-biased responses in BALB/c mice, Th1-biased responses in C57BL/6 mice, genetic heterogeneity in Kunming mice, and innate immune deficiencies in 129/Sv mice—may therefore influence protective efficacy [41,42]. In addition, age significantly moderated efficacy outcomes, with older mice appearing to exhibit stronger protection, which may reflect maturation of the immune system [43]. Together, these findings underscore the importance of controlling for host-related variables and standardizing experimental designs in future evaluations of S2308-derived gene-deletion vaccines.

The interpretation of the present synthesis should take the following constraints into account. First, substantial heterogeneity remained among the included studies even after subgroup analyses and meta-regression, indicating that unmeasured biological and methodological differences may have contributed to variation in the effect estimates. In addition, several subgroup estimates were informed by only a small number of studies or comparisons, which may have limited statistical precision and increased uncertainty around those estimates. Consequently, the combined findings should be viewed as suggestive rather than conclusive with respect to comparative vaccine efficacy. Moreover, this meta-analysis focused primarily on post-challenge splenic bacterial burden. Although splenic bacterial load is an important and commonly used indicator of bacterial clearance and vaccine-induced protection in mouse models of brucellosis, it does not fully capture all dimensions of protective immunity, such as histopathological changes, humoral and cellular immune responses, duration of protection, and safety. Finally, all included studies were conducted in mouse models. Although mouse models are valuable for the preliminary evaluation and comparative screening of candidate *Brucella* vaccines, these findings cannot be directly extrapolated to natural target hosts. Important differences exist between mice and livestock species in host physiology, immune architecture, infection route and challenge dose, disease manifestation, reproductive outcomes, and field-related safety requirements. Therefore, decreases in splenic bacterial load after challenge in mice should be viewed as preclinical indications of vaccine efficacy rather than direct demonstrations of protection in the intended animal hosts. Further standardized multi-dimensional studies of vaccine protection are required, which should evaluate post-challenge bacterial loads, histopathological lesions, humoral and cellular immune responses, safety profiles, and protective durability. Additionally, validation experiments in natural target hosts are necessary to verify the translational relevance of these vaccine candidates.

## 5. Conclusions

This meta-analysis provides a quantitative synthesis of the available mouse evidence comparing S2308-derived gene-deletion mutant vaccines with conventional *Brucella* vaccine strains. Based on post-challenge splenic bacterial burden, the available data suggest that S2308-derived gene-deletion mutant vaccines may provide protective efficacy broadly comparable to that of conventional vaccine strains in mouse models. In particular, deletion mutants targeting signal regulation-related genes were associated with lower post-challenge splenic bacterial loads than those targeting LPS synthesis-related pathways. However, these findings should be interpreted with caution due to high heterogeneity, insufficient data for subgroup analyses, dependence on a single primary endpoint, and exclusive employment of mouse models. Further standardized studies using broader protective endpoints and natural target hosts are needed to confirm the translational relevance of these vaccine candidates.

## Figures and Tables

**Table 1:** Characteristics of included comparison groups.

ID	First Author, Year	Ne ^a^	Nc ^b^	Gene Function	Deleted Gene	Mouse Strain, Age	Vaccination		Challenge		Interval ^h^
							Dose	Route	Dose	Route	
1	Cyntia, 2010 [13]	10	10	BM ^c^	*pgk*	C57BL/6, 6–9 w	1 × 10^5^ CFU	IP ^g^	1 × 10^6^ CFU	IP	12 w
2	Cyntia, 2010 [13]	10	10	BM	*pgk*	BALB/c, 6–9 w	1 × 10^5^ CFU	IP	1 × 10^6^ CFU	IP	12 w
3	Cyntia, 2010 [13]	10	10	BM	*pgk*	129/Sv, 6–9 w	1 × 10^5^ CFU	IP	1 × 10^6^ CFU	IP	12 w
4	D. Monreal, 2003 [14]	5	5	LPS-syn ^d^	*per*	BALB/c, 6 w	1 × 10^8^ CFU	IP	5 × 10^4^ CFU	IP	4 w
5	D. Monreal, 2003 [14]	5	5	LPS-syn	*wbkA*	BALB/c, 6 w	1 × 10^8^ CFU	IP	5 × 10^4^ CFU	IP	4 w
6	D. Monreal, 2003 [14]	5	5	LPS-syn	*manB*	BALB/c, 6 w	1 × 10^8^ CFU	IP	5 × 10^4^ CFU	IP	4 w
7	G.M.S, 2002 [15]	8	8	OM-TSP ^e^	*exsA*	BALB/c, 6–8 w	1 × 10^5^ CFU	IP	1 × 10^5^ CFU	IP	6 w
8	Job, 2015 [16]	8	8	OM-TSP	*omp19*	C57BL/6, 6–8 w	1 × 10^6^ CFU	IP	1 × 10^6^ CFU	IP	6 w
9	Job, 2015 [16]	8	8	OM-TSP	*mfp*	C57BL/6, 6–8 w	1 × 10^6^ CFU	IP	1 × 10^6^ CFU	IP	6 w
10	Juan, 2003 [17]	5	5	LPS-syn	*pgm*	BALB/c, 8–9 w	1 × 10^7^ CFU	IP	5 × 10^5^ CFU	IP	8 w
11	Junbo, 2014 [18]	10	10	BM	*ery*	BALB/c, 8 w	1 × 10^6^ CFU	IP	1 × 10^6^ CFU	IP	11 w
12	María, 2006 [19]	5	5	SR ^f^	*BvrR*	BALB/c, 7 w	1 × 10^8^ CFU	IP	1 × 10^5^ CFU	IP	4 w
13	María, 2006 [19]	5	5	SR	*wbkA&bvrS*	BALB/c, 7 w	1 × 10^8^ CFU	IP	1 × 10^5^ CFU	IP	4 w
14	María, 2006 [19]	5	5	LPS-syn	*wbkA*	BALB/c, 7 w	1 × 10^8^ CFU	IP	1 × 10^5^ CFU	IP	4 w
15	María, 2006 [19]	5	5	SR	*wbkA&bvrR*	BALB/c, 7 w	1 × 10^8^ CFU	IP	1 × 10^5^ CFU	IP	4 w
16	María, 2006 [19]	5	5	SR	*bvrS*	BALB/c, 7 w	1 × 10^8^ CFU	IP	1 × 10^5^ CFU	IP	4 w
17	Xiaowei Peng, 2019 [20]	12	12	SR	*clpP*	BALB/c, 4–6 w	1 × 10^5^ CFU	IP	2 × 10^5^ CFU	IP	8 w
18	Shuli Wang, 2020 [21]	5	5	SR	*gntR*	BALB/c, 6 w	1 × 10^6^ CFU	IP	1 × 10^6^ CFU	IP	15 w
19	Thaís, 2010 [22]	8	8	LPS-syn	*wbkC*	C57BL/6, 6–9 w	1 × 10^8^ CFU	IP	1 × 10^6^ CFU	IP	6 w
20	Zhen Wang, 2013 [23]	5	5	OM-TSP	*ATP/GTP*	BALB/c, 4–6 w	1 × 10^6^ CFU	IP	2 × 10^5^ CFU	IP	12 w
21	Huan Zhang, 2016 [24]	5	5	BM	*ery*	Kunming, 4–6 w	1 × 10^7^ CFU	IP	3 × 10^8^ CFU	IP	10 w
22	Huan Zhang, 2016 [24]	5	5	OM-TSP	*omp25*	Kunming, 4–6 w	1 × 10^7^ CFU	IP	3 × 10^8^ CFU	IP	10 w
23	Huan Zhang, 2016 [24]	5	5	LPS-syn	*wbkA*	Kunming, 4–6 w	1 × 10^7^ CFU	IP	3 × 10^8^ CFU	IP	10 w
24	Zhen Wang, 2013 [25]	5	5	LPS-syn	*wboA*	BALB/c, 4–6 w	1 × 10^6^ CFU	IP	2 × 10^5^ CFU	IP	14 w
25	Zhiqiang Li, 2015 [26]	10	10	LPS-syn	*wbkA*	BALB/c, 6 w	1 × 10^6^ CFU	IP	1 × 10^6^ CFU	IP	4 w
26	Zhiqiang Li, 2017 [27]	10	10	SR	*NodW*	BALB/c, 6 w	1 × 10^6^ CFU	IP	1 × 10^6^ CFU	IP	10 w
27	Zhiqiang Li, 2017 [27]	10	10	SR	*NodV*	BALB/c, 6 w	1 × 10^6^ CFU	IP	1 × 10^6^ CFU	IP	10 w
28	Zhiqiang Li, 2017 [27]	10	10	SR	*NodV&NodW*	BALB/c, 6 w	1 × 10^6^ CFU	IP	1 × 10^6^ CFU	IP	10 w

Note: ^a^ Ne = number of mice in the experimental group; ^b^ Nc = number of mice in the conventional-vaccine control group; ^c^ BM = basic metabolism; ^d^ LPS-syn = lipopolysaccharide synthesis; ^e^ OM-TSP = outer membrane-related transport and structural protein; ^f^ SR = signal regulation; ^g^ IP = intraperitoneal injection; ^h^ Interval = time interval between immunization and challenge; CFU = colony-forming units.

**Table 2:** Meta-regression analysis of factors associated with protective efficacy.

Variable	Coefficient (95% CI)	*p*-Value	R^2^ (%)	I^2^ (%)
Mouse Strain		0.04	14.25	93.04
C57BL/6	0.50 (−0.71, 1.72)	0.40		
Kunming	2.55 (0.63, 4.47)	0.01		
129/Sv	−1.01 (−3.25, 1.23)	0.36		
BALB/C	0.18 (−0.34, 0.71)	0.48		
Mouse Age		0.04	10.55	94.45
6–9 w	−0.96 (−1.88, −0.05)	0.04		
4–6 w	0.90 (0.23, 1.57)	0.01		
Gene Function		0.03	17.75	93.20
SR	−1.45 (−2.56, −0.34)	0.01		
BM	−1.16 (−2.46, 0.14)	0.07		
OM-TSP	−1.00 (−2.30, 0.31)	0.09		
LPS-syn	1.24 (0.44, 2.04)	<0.01		
Challenge Dose		<0.01	47.57	91.24
10^4^ CFU	2.39 (1.05, 3.73)	<0.01		
105 CFU	−0.03 (−0.82, 0.76)	0.94		
10^6^ CFU	0.01 (−0.51, 0.53)	0.97		
10^8^ CFU	2.72 (0.97, 4.48)	<0.01		
No. of deleted genes		0.02	18.77	94.44
Single	0.56 (0.09, 1.02)	0.02		
Double	−1.69 (−3.08, −0.31)	<0.01		

Note: CI = confidence interval; CFU = colony-forming units; R^2^ = proportion of between-study variance explained; I^2^ = inconsistency statistic.

## Data Availability

The original contributions presented in this study are included in the article/Supplementary Materials. Further inquiries can be directed to the corresponding authors.

## Supplementary Materials

The following supporting information can be downloaded at: https://www.mdpi.com/article/10.3390/microorganisms14071419/s1, Table S1. Search strategy. Figure S1. Forest plot for subgroup analysis by gene function. Figure S2. Forest plot for subgroup analysis by mouse strain. Figure S3. Forest plot for subgroup analysis by mouse age. Figure S4. Forest plot for subgroup analysis by number of deleted genes. Figure S5. Forest plot for subgroup analysis by challenge dose. Figure S6. Forest plot for subgroup analysis by immunization dose.
