# Supplementary material for: A Meta-Analysis of the Protective Efficacy of Brucella abortus S2308 Gene-Deletion Mutant Vaccines Compared to Conventional Vaccines in Mice
Source: Microorganisms. 2026 Jun 29;14(7):1419. doi: 10.3390/microorganisms14071419 (PMC13413911; doi:10.3390/microorganisms14071419)
Supplement: Supplementary file 1 [file microorganisms-14-01419-s001.zip › microorganisms-4354943-supplementary.pdf]

microorganisms MDPI

Supplementary Materials

Table S1. Search strategy.

Database	Keywords
PubMed(364)	((*Brucella*[MeSH] OR *Brucella*[tiab] OR Brucellosis[MeSH] OR Brucellosis[tiab] OR "*Brucella abortus*"[tiab] OR "*Brucella melitensis*"[tiab] OR "*Brucella suis*"[tiab]) AND (vaccin*[tiab] OR immuni*[tiab] OR "immune response"[tiab] OR inoculat*[tiab] OR immunogen*[tiab]) AND (delet*[tiab] OR knockout[tiab] OR "gene deletion"[MeSH] OR "gene knock-out"[tiab] OR mutant*[tiab] OR mutation[tiab] OR "genetic engineering"[MeSH] OR "genetically modified"[tiab] OR attenuated[tiab]) AND (efficac*[tiab] OR effectiveness[tiab] OR safety[tiab] OR "adverse effects"[tiab] OR protect*[tiab] OR immunogen*[tiab] OR "antibody response"[tiab] OR "cellular immunity"[tiab]))
Web of Science(802)	(*Brucella* OR Brucellosis OR "*Brucella abortus*" OR "*Brucella melitensis*" OR "*Brucella suis*") AND (vaccin* OR immuni* OR "immune response" OR inoculat* OR immunogen* OR "antibody response" OR "cellular immune" OR "protective immunity" OR "dose response") AND (delet* OR knockout OR "gene deletion" OR "gene knock-out" OR mutant* OR mutation OR "genetic engineering" OR "genetically modified" OR attenuated) AND (efficac* OR effectiveness OR safety OR "adverse effects" OR protect* OR immunogen* OR "cellular immunity")<br>**Note: On the left side of the page, check the "Article" and "Dissertation Thesis" options under "Document Types".**
ScienceDirect(1104)	(("*Brucella*" OR "Brucellosis") AND ("vaccine" OR "immunization" OR "immunogen") AND ("gene deletion" OR "gene knockout" OR "genetically modified" OR "attenuated"))<br>**Note: On the left side of the page, check the "Research Articles" option under "Document Type".**
CNKI(136)	(*Brucella* OR *Brucella* spp.) AND (gene deletion OR gene knockout OR gene mutation) AND (immunity OR protection OR safety OR immune protection OR evaluation)
Wanfang(159)	(*Brucella* OR *Brucella* spp.) AND (gene deletion OR gene knockout OR gene mutation) AND (immunity OR protection OR safety OR immune protection OR evaluation)
VIP(21)	(*Brucella* OR Brucellosis) AND (gene deletion OR gene knockout OR gene mutation) AND (immunity OR protection OR safety OR immune protection OR evaluation)

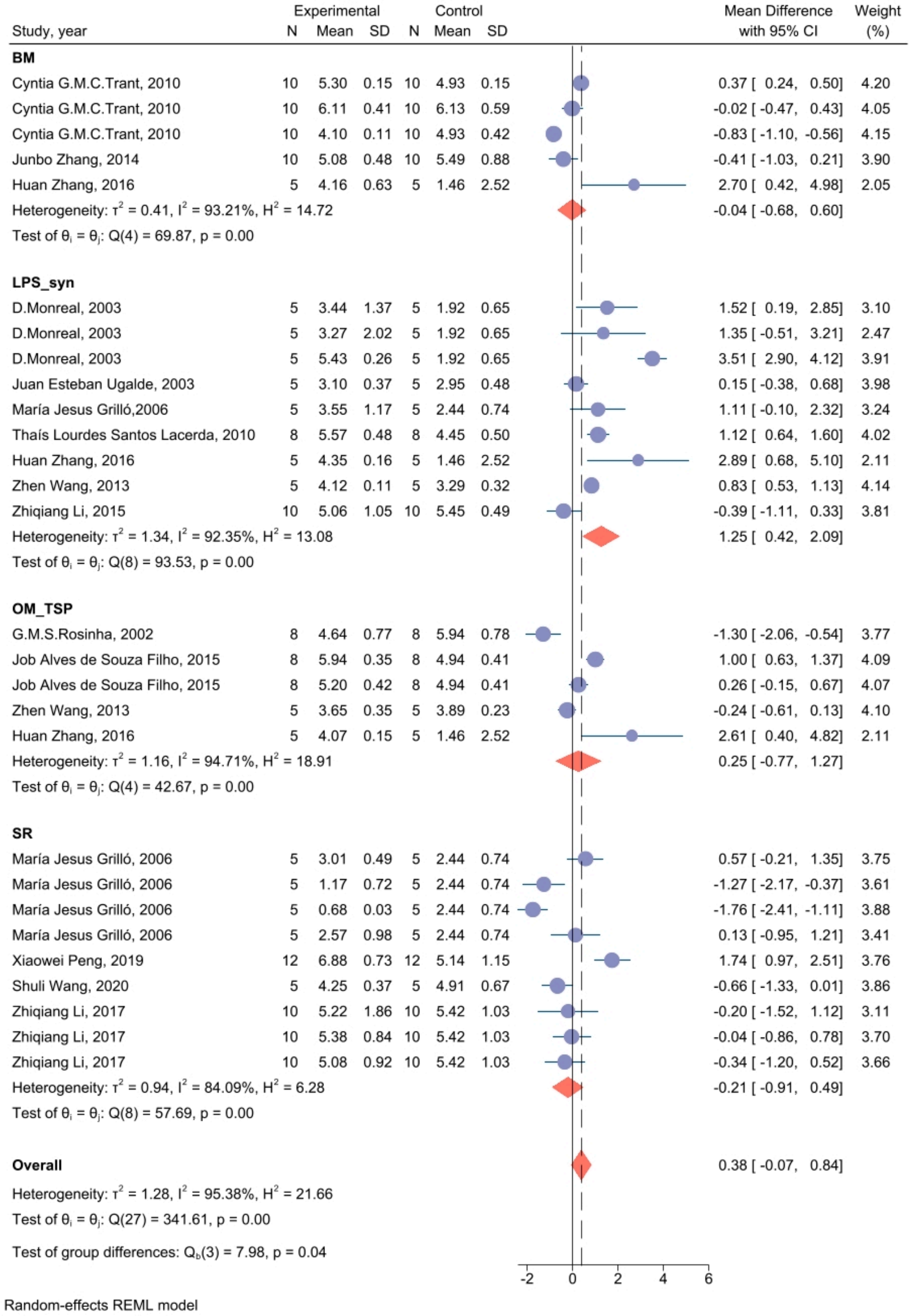

Figure S1. Forest plot for subgroup analysis by gene function.

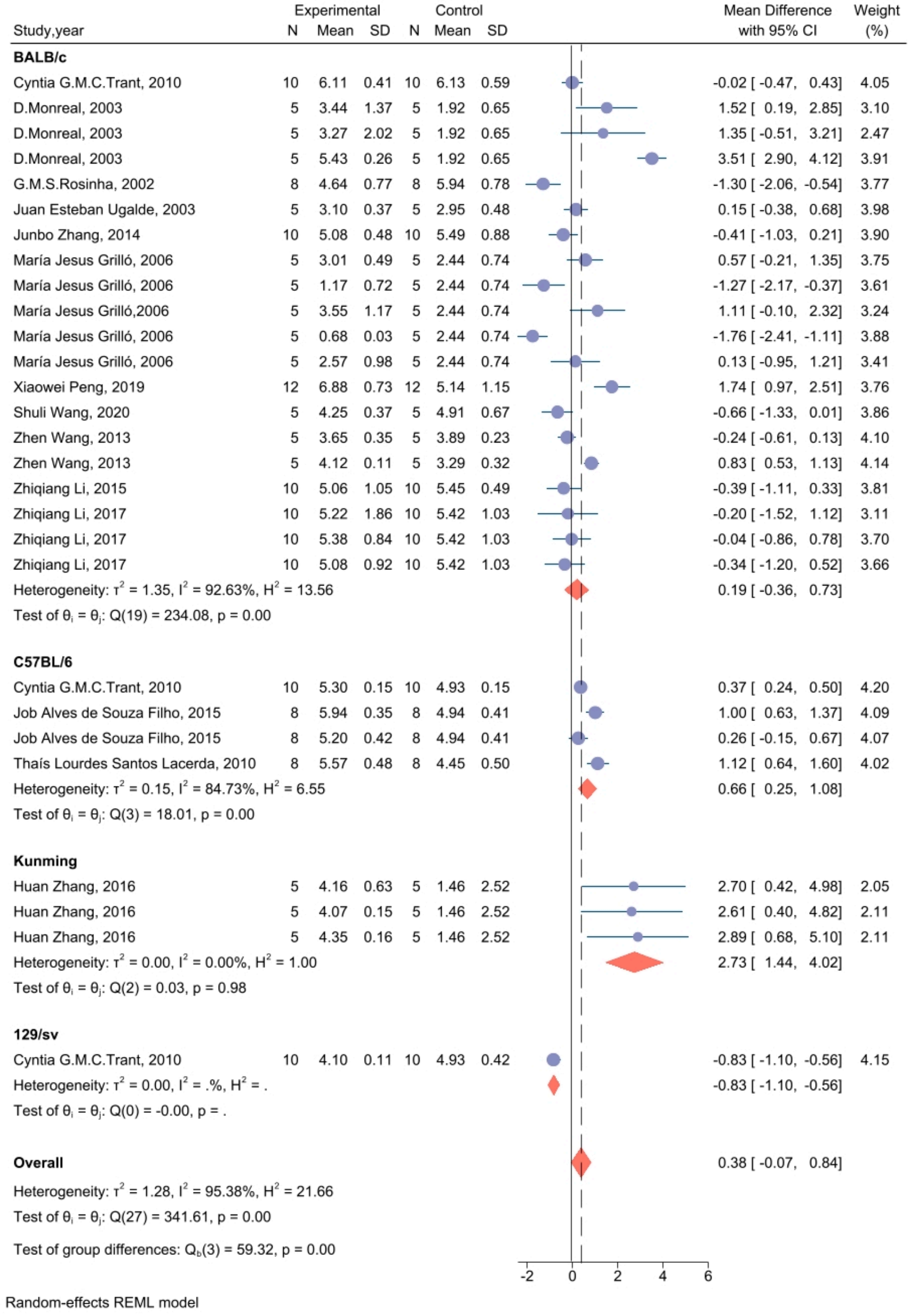

Figure S2. Forest plot for subgroup analysis by mouse strain.

Study, year	Experimental N	Experimental Mean	Experimental SD	Control N	Control Mean	Control SD	Mean Difference with 95% CI	Weight (%)
**4-6w**								
D.Monreal, 2003	5	3.44	1.37	5	1.92	0.65	1.52 [0.19, 2.85]	3.10
D.Monreal, 2003	5	3.27	2.02	5	1.92	0.65	1.35 [-0.51, 3.21]	2.47
D.Monreal, 2003	5	5.43	0.26	5	1.92	0.65	3.51 [2.90, 4.12]	3.91
Xiaowei Peng, 2019	12	6.88	0.73	12	5.14	1.15	1.74 [0.97, 2.51]	3.76
Shuli Wang, 2020	5	4.25	0.37	5	4.91	0.67	-0.66 [-1.33, 0.01]	3.86
Zhen Wang, 2013	5	3.65	0.35	5	3.89	0.23	-0.24 [-0.61, 0.13]	4.10
Huan Zhang, 2016	5	4.16	0.63	5	1.46	2.52	2.70 [0.42, 4.98]	2.05
Huan Zhang, 2016	5	4.07	0.15	5	1.46	2.52	2.61 [0.40, 4.82]	2.11
Huan Zhang, 2016	5	4.35	0.16	5	1.46	2.52	2.89 [0.68, 5.10]	2.11
Zhen Wang, 2013	5	4.12	0.11	5	3.29	0.32	0.83 [0.53, 1.13]	4.14
Zhiqiang Li, 2015	10	5.06	1.05	10	5.45	0.49	-0.39 [-1.11, 0.33]	3.81
Zhiqiang Li, 2017	10	5.22	1.86	10	5.42	1.03	-0.20 [-1.52, 1.12]	3.11
Zhiqiang Li, 2017	10	5.38	0.84	10	5.42	1.03	-0.04 [-0.86, 0.78]	3.70
Zhiqiang Li, 2017	10	5.08	0.92	10	5.42	1.03	-0.34 [-1.20, 0.52]	3.66
Heterogeneity: $\tau^2 = 1.69$, $I^2 = 92.76\%$, $H^2 = 13.81$							0.94 [0.19, 1.69]	
Test of $\theta_i = \theta_j$: Q(13) = 159.40, p = 0.00								
**6-9w**								
Cyntia G.M.C.Trant, 2010	10	5.30	0.15	10	4.93	0.15	0.37 [0.24, 0.50]	4.20
Cyntia G.M.C.Trant, 2010	10	6.11	0.41	10	6.13	0.59	-0.02 [-0.47, 0.43]	4.05
Cyntia G.M.C.Trant, 2010	10	4.10	0.11	10	4.93	0.42	-0.83 [-1.10, -0.56]	4.15
G.M.S.Rosinha, 2002	8	4.64	0.77	8	5.94	0.78	-1.30 [-2.06, -0.54]	3.77
Job Alves de Souza Filho, 2015	8	5.94	0.35	8	4.94	0.41	1.00 [0.63, 1.37]	4.09
Job Alves de Souza Filho, 2015	8	5.20	0.42	8	4.94	0.41	0.26 [-0.15, 0.67]	4.07
Juan Esteban Ugalde, 2003	5	3.10	0.37	5	2.95	0.48	0.15 [-0.38, 0.68]	3.98
Junbo Zhang, 2014	10	5.08	0.48	10	5.49	0.88	-0.41 [-1.03, 0.21]	3.90
María Jesus Grilló, 2006	5	3.01	0.49	5	2.44	0.74	0.57 [-0.21, 1.35]	3.75
María Jesus Grilló, 2006	5	1.17	0.72	5	2.44	0.74	-1.27 [-2.17, -0.37]	3.61
María Jesus Grilló,2006	5	3.55	1.17	5	2.44	0.74	1.11 [-0.10, 2.32]	3.24
María Jesus Grilló, 2006	5	0.68	0.03	5	2.44	0.74	-1.76 [-2.41, -1.11]	3.88
María Jesus Grilló, 2006	5	2.57	0.98	5	2.44	0.74	0.13 [-0.95, 1.21]	3.41
Thaís Lourdes Santos Lacerda, 2010	8	5.57	0.48	8	4.45	0.50	1.12 [0.64, 1.60]	4.02
Heterogeneity: $\tau^2 = 0.72$, $I^2 = 94.12\%$, $H^2 = 17.00$							-0.06 [-0.54, 0.42]	
Test of $\theta_i = \theta_j$: Q(13) = 161.46, p = 0.00								
**Overall**							0.38 [-0.07, 0.84]	
Heterogeneity: $\tau^2 = 1.28$, $I^2 = 95.38\%$, $H^2 = 21.66$								
Test of $\theta_i = \theta_j$: Q(27) = 341.61, p = 0.00								
Test of group differences: $Q_b(1) = 4.84$, p = 0.03								

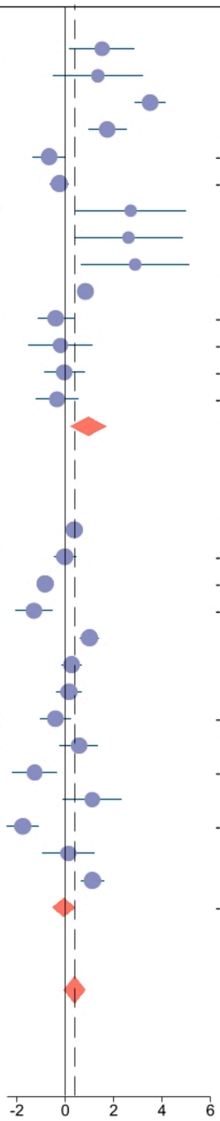

Random-effects REML model

Figure S3. Forest plot for subgroup analysis by mouse age.

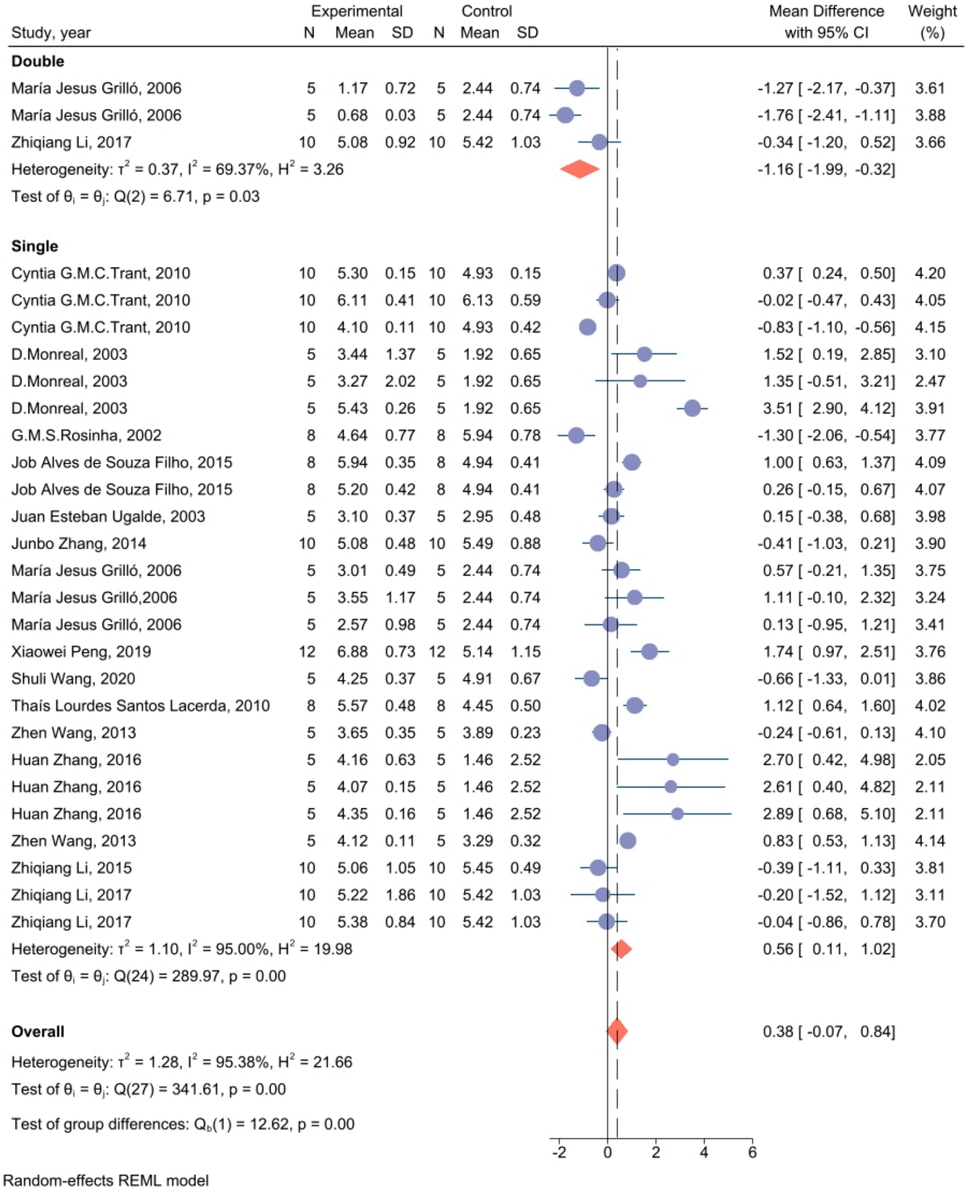

Figure S4. Forest plot for subgroup analysis by number of deleted genes.

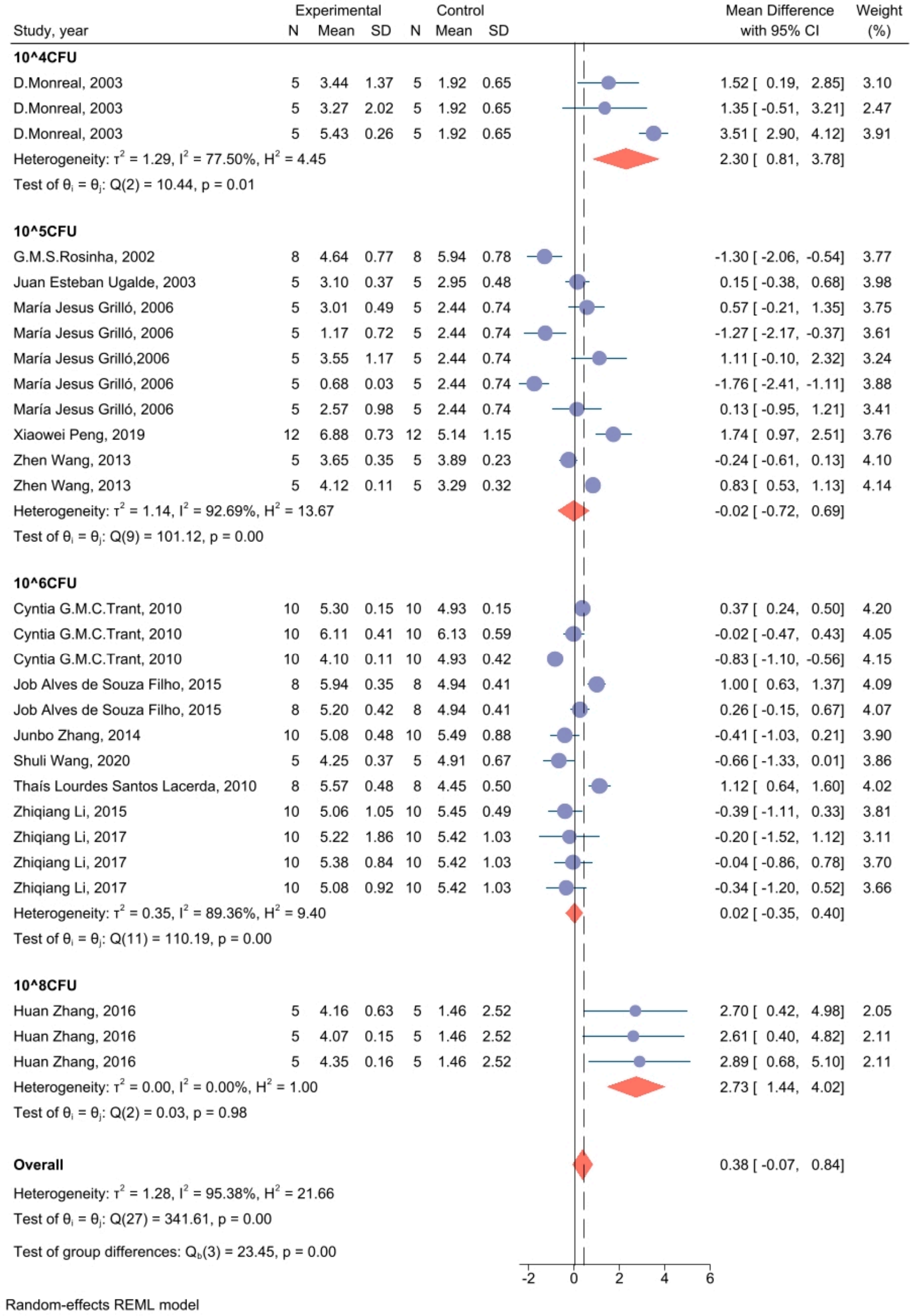

Figure S5. Forest plot for subgroup analysis by challenge dose.

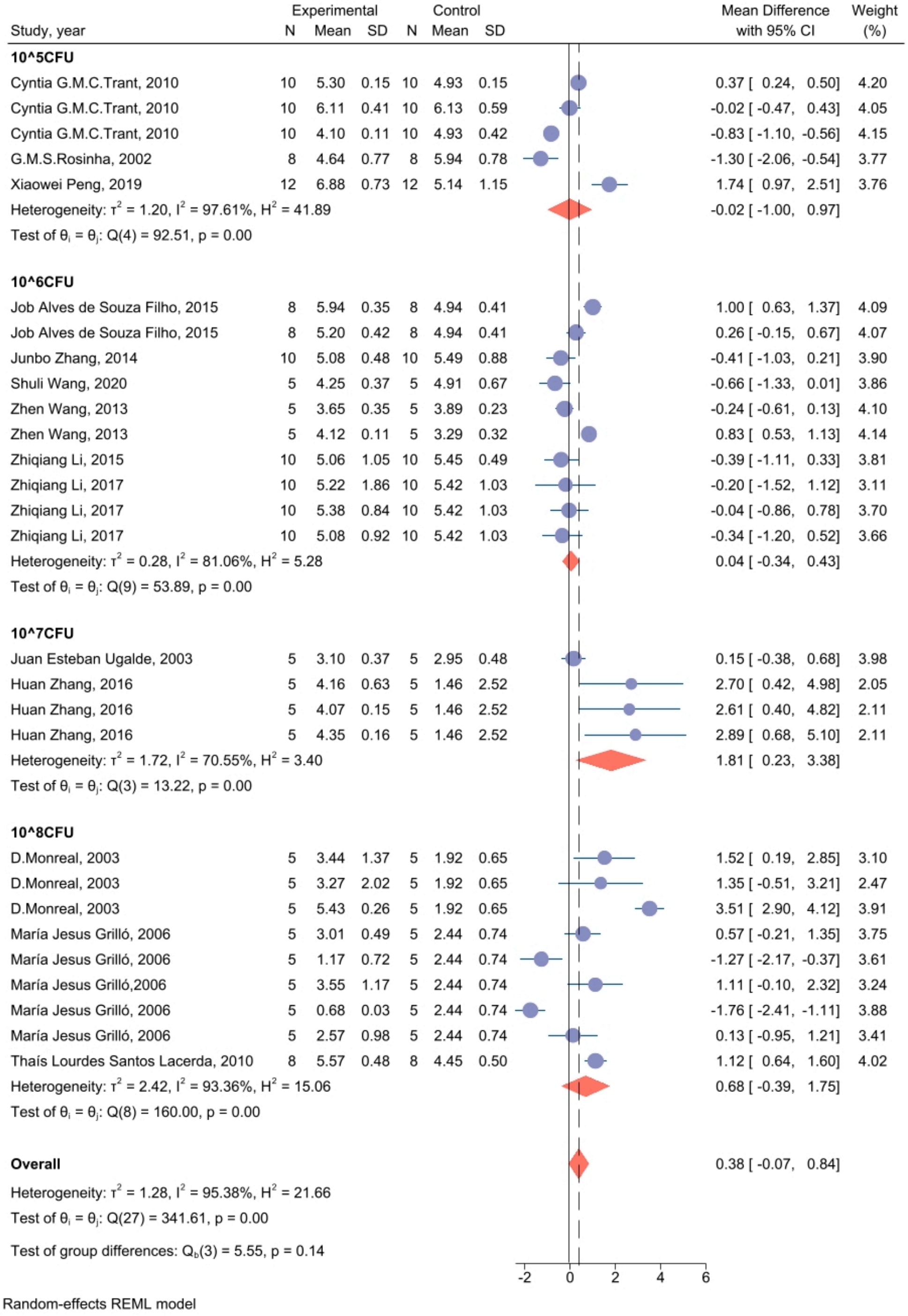

Figure S6. Forest plot for subgroup analysis by immunization dose.
